# IL-10’s contextual role in cytotoxic antitumor immunity: evidence, controversies, and therapeutic translation

**DOI:** 10.3389/fimmu.2026.1898790

**Published:** 2026-09-07

**Authors:** Minchuan Zhang, Qi-Jing Li, Kong Peng Lam, Shengli Xu

**Affiliations:** 1 Singapore Immunology Network, Agency for Science, Technology, and Research, Singapore, Singapore; 2 Institute of Molecular and Cell Biology, Agency for Science, Technology, and Research, Singapore, Singapore; 3 Department of Microbiology and Immunology, Yong Loo Lin School of Medicine, National University of Singapore, Singapore, Singapore; 4 Department of Physiology, Yong Loo Lin School of Medicine, National University of Singapore, Singapore, Singapore

**Keywords:** cancer immunotherapy, CAR-T cell, CD8+ T cells, IL-10 receptor blockade, interleukin-10, metabolic reprogramming, natural killer cells, T-cell exhaustion

## Abstract

Interleukin-10 (IL-10) is classically defined as a potent anti-inflammatory cytokine that protects tissues by constraining myeloid activation and limiting inflammatory cytokine production. Yet, accumulating evidence indicates that, in defined contexts, IL-10 can also promote antitumor immunity by enhancing the fitness and cytotoxic programs of CD8^+^ T cells and natural killer (NK) cells. Conversely, in other tumor contexts, IL-10 may reinforce immunosuppressive myeloid and regulatory programs, and the factors that determine these divergent outcomes remain incompletely understood. Although these effects converge on a common JAK1/TYK2–STAT3 signaling hub, the downstream biological outcomes are combinatorially shaped by the identity, activation, and chromatin state of the responding cell, co-engaged pathways, including STAT1, mTORC1, and NF-κB/AP, receptor abundance, feedback regulation, and the surrounding microenvironment. Recent studies suggest IL-10 can sustain cytotoxic lymphocyte function under tumor microenvironmental stress through effector programming, restraint of terminal exhaustion, mitochondrial metabolic reprogramming, and remodeling of the tumor microenvironment (TME). However, several of these mechanisms remain supported primarily by limited preclinical models and correlative human data. These advances have promoted the development of pharmacokinetically optimized IL-10 variants, tumor-targeted immunocytokines, surrogate bispecific IL-10 receptor agonists, and IL-10–armored adoptive cell therapies. Although several approaches have shown immune activation and preliminary antitumor activity in early-phase studies, pegilodecakin—the only IL-10-based agent to complete a randomized phase III oncology trial—did not improve survival in gemcitabine-refractory pancreatic cancer, underscoring the gap between pharmacodynamic activity and established clinical benefit. In this review, we synthesize mechanistic and translational advances with conflicting and cautionary evidence, discuss safety considerations associated with IL-10-based agents and IL-10-armored cell therapies, and propose a context-dependent framework for their further development based on tumor and microenvironmental features, spatiotemporal control, rational combination strategies, and candidate biomarkers.

## 1 Introduction

IL-10 has historically been viewed as a prototypical anti-inflammatory cytokine that protects host tissues by limiting the magnitude and duration of immune activation (1). Acting predominantly on antigen-presenting cells, IL-10 suppresses pro-inflammatory cytokine production, reduces antigen presentation and co-stimulatory signaling, and thereby restrains downstream effector responses while promoting resolution of inflammation (2, 3). Accordingly, IL-10 biology has been studied extensively in the contexts of infection, autoimmunity, and chronic inflammatory disease.

Accumulating evidence, however, indicates that IL-10 biology extends beyond these canonical anti-inflammatory functions, particularly in cancer immunity and immunotherapy. Under defined conditions, IL-10 can augment the effector functions of CD8^+^ T cells and NK cells – two cytotoxic lymphocyte populations central to antitumor immunity (4–9). This pro-cytotoxic activity coexists with substantial evidence that IL-10/IL-10R signaling can also support protumor and immunosuppressive programs in other cellular and tumor contexts. Elevated IL-10 has been linked to reduced CD8^+^ T-cell antigen sensitivity through N-glycan branching (10), reinforcement of tumor-associated macrophage (TAM)-mediated drug resistance in multiple myeloma – a process reversed by IL-10R blockade (11) – and, more broadly, a “paradoxical” role in which its net effect on tumor growth varies across tumor types and immune contexts (12, 13). Reconciling these opposing observations, rather than privileging one side of literature, is a central aim of this review.

A key principle emerging from these findings is that the biological outcome of IL-10 signaling is highly context dependent. Its effects are shaped by dose and timing, the identity and activation state of the responding cell, IL-10R expression, and local microenvironmental cues (2, 14, 15). IL-10 should therefore be viewed neither simply as an anti-inflammatory cytokine nor as an enhancer of antitumor immunity, but as a pleiotropic immunoregulatory cytokine whose effects depend on the cellular and tissue context in which signaling occurs. Consequently, therapeutic manipulation of this pathway — whether through IL-10 agonism, targeted delivery, or IL-10R blockade — must be matched to the immunological context of the tumor being treated.

Building on this context-dependent view, the field has begun to move beyond descriptive concept of IL-10’s “dual role” toward a more mechanistic understanding of how these divergent effects arise. Recent studies have started to define how IL-10 directly supports cytotoxic lymphocyte differentiation, survival, and effector function (9, 16, 17), while reprogramming cellular metabolism to sustain these responses under the tumor microenvironment (TME) stress such as nutrient deprivation and hypoxia (5, 18). These advances suggest that the functional outcome of IL-10 signaling is determined not by a single downstream pathway in isolation, but by the interaction between a common signaling framework and the molecular state of the responding cell.

In parallel, these mechanistic insights have catalyzed translational strategies that seek to harness or redirect IL-10 biology for cancer immunotherapies (8, 19–22). These approaches include engineered IL-10 variants, tumor-targeted delivery systems, surrogate receptor agonists, combination regimens, and IL-10–armored adoptive cell therapies. Early clinical studies have reported evidence of immune activation and preliminary antitumor activity in selected settings (5, 9). However, the one completed randomized phase III trial of an IL-10–based cancer therapy did not demonstrate a survival benefit (23), underscoring the gap between pharmacodynamic immune activation and proven clinical efficacy.

In this review, we integrate the evidence for both the cytotoxicity-enhancing and immunosuppressive functions of IL-10 in cancer and examine their implications for immunotherapy. We first discuss advances linking IL-10/IL-10R signaling to cytotoxic lymphocyte function, metabolic fitness, and persistence, alongside evidence that the same pathway can reinforce immunosuppressive myeloid and regulatory programs. We then propose that these apparently opposing effects represent context-dependent outputs of a shared signaling hub acting in distinct cellular states and shaped by additional pathway inputs, rather than an axis that is intrinsically beneficial or harmful. Finally, we examine emerging therapeutic strategies and propose a decision framework for identifying the settings in which IL-10 agonism, targeted delivery, or IL-10R blockade may be most appropriate, together with the biomarker and safety requirements needed for rigorous clinical translation.

## 2 IL-10 and IL-10R: expression, structure, and signaling

IL-10 was originally identified as cytokine synthesis inhibitory factor (CSIF) produced by T helper 2 (Th2) cells. It is now clear, however, that IL-10 is produced by a broad range of myeloid and lymphoid populations and, in some contexts, by non-hematopoietic cells, including epithelial and stromal cells (1). Among immune cells, macrophages and dendritic cells (DCs) are major sources of IL-10, with additional production reported in neutrophils, NK cells, B cells - including regulatory B-cell subsets - multiple CD4^+^ T-cell populations, including Foxp3^+^ regulatory T cells (Tregs), and activated CD8^+^ T cells (1, 3, 24–34). This broad and highly stimulus-dependent expression pattern underlies IL-10’s central role in constraining inflammation and maintaining tissue homeostasis across diverse physiological and pathological settings.

Murine and human IL-10 are both 160 amino acids proteins that characteristically form non-covalently associated homodimers through domain swapping of α-helical segments between monomers (1, 35). This dimeric architecture stabilizes the cytokine and establishes the receptor-binding geometry required for productive engagement of the IL-10 receptor (IL-10R). Notably, engineered monomeric IL-10 variants nevertheless retain measurable biological activity, indicating that dimerization enhances receptor assembly and signaling efficiency but is not absolutely required for function (36).

The IL-10R belongs to the class II cytokine receptor family, whose members lack the canonical Trp-Ser-xTrp-Ser motif found in the extracellular domains of class I cytokine receptors (1). The IL-10R is composed of two chains: the ligand-binding α chain, IL-10Rα (IL-10RA, CD210a, or IL-10R1) and the accessory β chain, IL-10Rβ, (IL-10RB, CD210b, or IL-10R2). IL-10Rβ is broadly expressed and serves as a shared low-affinity subunit for several class II cytokines, including IL-10, IL-22, IL-26, and the type III interferons IL-28 and IL-29 (IFN-λ), which collectively contribute to host defense and barrier immunity (37). In contrast, the IL-10Rα chain is primarily expressed on leukocytes and binds IL-10 with high affinity (37, 38). It therefore confers IL-10-binding specificity and restricting robust IL-10 responsiveness largely to hematopoietic cells (1, 38). In the absence of ligand, the two receptor chains are largely unassembled, while their cytoplasmic tails are constitutively associated with Janus kinases (JAK)- JAK1 with IL-10Rα and tyrosine kinase 2 (TYK2) with IL-10Rβ.

**Figure 1:**
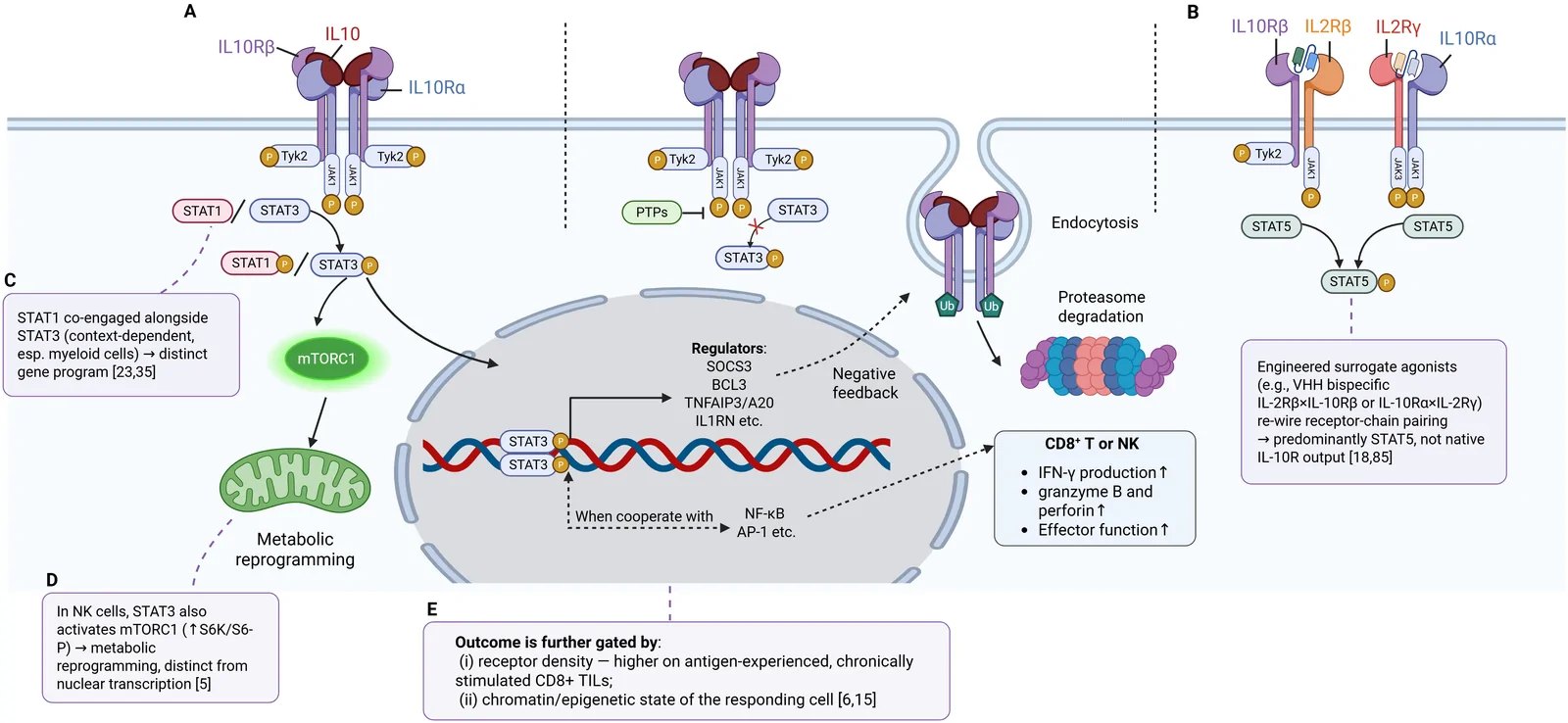
Structure and signaling of the IL-10 receptor. **(A)** The IL-10 receptor comprises a ligand-binding α chain (IL-10Rα) and a shared accessory β chain (IL-10Rβ). Binding of an IL-10 homodimer drives assembly of a 2:2:2 hetero-hexameric complex containing one IL-10 dimer, two IL-10Rα chains, and two IL-10Rβ chains. The complex assembly juxtaposes the receptor-associated Janus kinases JAK1 and TYK2, triggering their activation and subsequent STAT3 phosphorylation. IL-10R signaling is constrained by several negative-feedback mechanisms, including induction of SOCS proteins and phosphatases (PTPs), which reduce JAK–STAT phosphorylation, and receptor downregulation through ubiquitination and endocytosis. In activated NK cells and CD8^+^ T cells (lower right), STAT3 may cooperate with transcription factors such as NF-κB and AP-1 to promote cytotoxic effector programs, including expression of IFN-γ, granzyme B, and perforin. **(B)** Engineered surrogate agonists, such as VHH-based IL-2Rβ×IL-10Rβ or IL-10Rα×IL-2Rγ bispecific, reconfigure receptor-chain pairing and induce STAT5-biased signaling rather than the STAT3-dominant output of native IL-10R engagement. **(C)** STAT1 may be co-activated with STAT3 in a context-dependent manner, particularly in myeloid cells, thereby producing distinct signaling outputs. **(D)** In NK cells, STAT3 also activates mTORC1 as indicated by increased S6K and S6 phosphorylation, to support metabolic reprogramming and cytotoxic function. **(E)** Signaling strength and transcriptional output are further shaped by receptor abundance, which is elevated on antigen-experienced, chronically stimulated CD8^+^ tumor-infiltrating lymphocyte (TILs), and by the pre-existing chromatin and epigenetic state of the responding cell. Together, panels **(B–E)** illustrate that IL-10R signaling outcomes are combinatorially determined by receptor-chain pairing, co-engaged pathways, signaling amplitude, and cellular state rather than by STAT3 phosphorylation alone.

Ligand binding drives formation of a signaling-competent heterohexameric complex comprising one IL-10 homodimer, two IL-10Rα chains, and two IL-10Rβ chains (**Figure 1**). Assembly proceeds stepwise. Dimeric IL-10 first engages the high-affinity IL-10Rα subunits, thereby increasing its apparent affinity for IL-10Rβ and promoting recruitment of the accessory chains to form the complete 2:2:2 complex (3, 39). This assembly juxtaposes the receptor-associated JAK1 (bound to IL-10Rα) and TYK2 (bound to IL-10Rβ), enabling their mutual activation and initiating downstream JAK– STAT signaling.

Activated JAK1 and TYK2 phosphorylate tyrosine residues within the IL-10Rα cytoplasmic tail, creating docking sites for STAT proteins. STAT3 is the dominant effector, although STAT1 can also be engaged in a context-dependent manner (2, 40). Following phosphorylation, STAT3 dimerizes and translocates to the nucleus. In myeloid cells, it induces a characteristic anti-inflammatory transcriptional program that includes suppressor of cytokine signaling 3 (SOCS3), BCL3, TNFAIP3/A20, IL1RN, and other regulators that dampen NF-κB and MAPK signaling. This program selectively suppresses the production of pro-inflammatory cytokines, such as TNF-α, IL-1β, IL-6, IL-12 and IL-23 (41, 42). Consistent with this central role, genetic deletion or functional disruption of STAT3 in macrophages and neutrophils abrogates IL-10 responsiveness and compromises IL-10–mediated suppression of inflammatory cytokine production (1, 43).

IL-10R signaling is also subject to multiple layers of negative regulation that limit its amplitude and duration. These include induction of SOCS proteins, particularly SOCS3 and SOCS1; dephosphorylation by phosphatases such as Src homology region 2 domain-containing phosphatase-1 (SHP-1), receptor ubiquitination and downregulation, including beta-transducin repeat-containing protein (βTrCP)-mediated mechanisms; and regulation of endocytic trafficking by proteins such as ATG16L1 (44). These feedback mechanisms prevent prolonged or excessive immunosuppression and have direct implications for therapeutic design, because the dose, duration, and cellular distribution of IL-10 exposure may critically influence whether the signaling favors anti-inflammatory or cytotoxicity-enhancing outcomes.

Although the canonical JAK1/TYK2–STAT3 pathway explains many established effects of IL-10, it does not fully account for the diversity of biological responses observed across cell types. In particular, the divergent functions of IL-10 should not be attributed to STAT3 activation in isolation. We instead propose that IL-10R output is determined combinatorially by the receptor chains and kinases engaged, the signaling pathways activated in parallel, the abundance and duration of receptor signaling, and the transcriptional and epigenetic state of the responding cells.

Several lines of evidence support this broader model. First, STAT1 can be activated alongside STAT3, particularly in myeloid cells, and can redirect the transcriptional response toward a distinct gene program (28, 40). Second, engineered surrogate agonists that re-wire IL-10R-chain pairing — for example, VHH-based bispecific IL-2Rβ×IL-10Rβ or IL-10Rα×IL-2Rγ bispecific constructs — predominantly activate STAT5. These studies demonstrated that signaling output depends heavily on which receptor-associated JAK kinases are physically juxtaposed, rather than being on invariant consequence of IL-10Rα engagement (22, 45). Third, in NK cells, IL-10–driven STAT3 activation engages mTORC1, as reflected by increased S6K and S6 phosphorylation. This STAT3–mTORC1 axis, rather than STAT3 alone, is required for the metabolic reprogramming that supports enhanced NK cell cytotoxicity (5).

Fourth, cytotoxic effector genes are regulated in part by NF-κB and AP-1. Functional AP-1 and NF-κB elements control granzyme B transcription in antigen-experienced CD8^+^ T cells and in human NK and T cells, respectively (46, 47), while STAT3 can cooperate with NF-κB at shared target genes in other contexts (48). Although direct STAT3–NF-κB/AP-1 cooperation in cytotoxic lymphocytes remains unproven, it offers a plausible explanation for why IL-10–induced STAT3 activation may favor cytotoxic rather than anti-inflammatory gene programs in these cells.

Fifth, the amplitude and duration of IL-10R signaling are shaped by receptor abundance and feedback regulation. IL-10R expression varies with cellular activation state and is markedly higher on antigen-experienced, chronically stimulated CD8^+^ tumor-infiltrating lymphocytes (TIL) than on naïve lymphocytes (6, 19). Together with the negative-feedback mechanisms described above, this variation in receptor density is likely to influence signaling strength, persistence, and ultimately biological outcome.

Finally, the pre-existing chromatin and epigenetic state of the responding cell likely determines which genes are accessible to activated STAT3. Genome-wide mapping shows that STAT3 binds predominantly within pre-existing, nucleosome-depleted chromatin rather than opening new regulatory regions (49). Moreover, exhausted CD8^+^ T cells acquire a distinct and stable chromatin accessibility landscape that differs from those of naïve and memory cells and is not fully reversed by checkpoint blockade (50). Together, these findings provide a plausible structural basis for why the same STAT3 phosphorylation event may produce different transcriptional outcomes in resting versus antigen-experienced or partially exhausted CD8^+^ T cells. We return to this combinatorial view of IL-10R signaling in the Conclusions and Future Directions.

## 3 Canonical anti-inflammatory functions of IL-10

**Figure 2:**
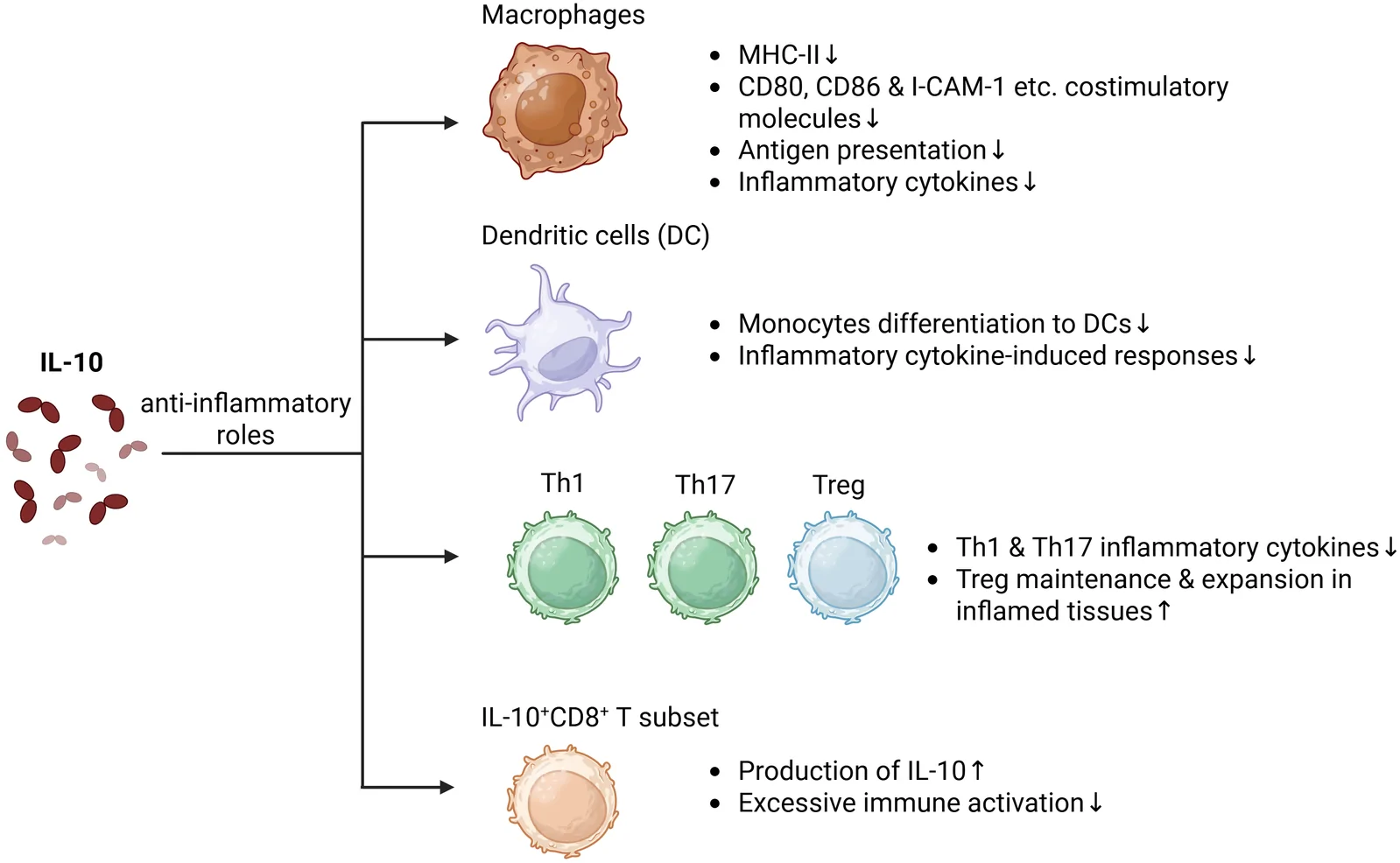
Conventional anti-inflammatory functions of IL-10 across immune cell types. IL-10 restrains inflammation through coordinated effects on innate and adaptive immune cells. In macrophages, IL-10 downregulates MHC class II, the co-stimulatory molecules CD80 and CD86 and the adhesion molecule ICAM-1, thereby suppressing antigen presentation and pro-inflammatory cytokine production. In the dendritic cell (DC) compartment, IL-10 inhibits monocyte-to-DC differentiation and attenuates DC responsiveness to inflammatory stimuli. In helper T cells, IL-10 limits inflammatory cytokine secretion by Th1 and Th17 cells, whereas in regulatory T (Treg) cells it supports their maintenance and expansion within inflamed tissues. In IL-10-producing CD8^+^ T-cell subsets, autocrine These canonical suppressive effects are particularly relevant in inflammatory and myeloid-dominant tumor contexts and provide the biological rationale for settings in which IL-10R blockade may be favored over agonism (Section 4.6; **Figure 3**).

IL-10 is classically viewed as an immunosuppressive cytokine that restrains inflammation through coordinated effects on both innate and adaptive immune compartments (**Figure 2**). In macrophages, IL-10 dampens antigen presentation by reducing major histocompatibility complex class II (MHC-II) expression and downregulates co-stimulatory molecules such as CD80 and CD86, thereby limiting T-cell priming (51). IL-10 also shapes myeloid differentiation and function by inhibiting monocyte differentiation into DCs (31) and counteracting inflammatory programs driven by mediators such as TNF-α, IL-6, and IFN-γ (27, 52). Collectively, these effects suppress pro-inflammatory cytokine production, interrupt the feed-forward loops that sustain innate immune activation, and limit tissue inflammation.

IL-10 exerts parallel regulatory effects on adaptive immunity. In T cells, IL-10R–STAT3 signaling activates transcriptional programs that constrain inflammatory cytokine production and limit immunopathology. In Th1 cells, this pathway restrains type I inflammatory response and the associated tissue damage (51). Similar suppressive effects have been described in Th17 cells, contributing to the containment of pathogenic Th17-driven inflammation (53). Treg cells are both important sources and targets of IL-10. IL-10 signaling induces STAT3 phosphorylation in Tregs and can support their maintenance or expansion within inflamed tissues, thereby reinforcing local immune regulation (54).

IL-10-mediated suppression is not restricted to dedicated regulatory populations. A subset of CD8^+^ T cells can produce substantial IL-10 at sites of infection, including the lung and brain, establishing autocrine and paracrine negative-feedback loops that tempers excessive immune activation and reduce collateral tissue damage (10, 34). Through these complementary actions across myeloid and lymphoid compartments, IL-10 calibrates the magnitude and duration of immune responses, promotes resolution, and preserves tissue integrity.

The same regulatory properties that protect tissues from immunopathology can, however, be exploited by pathogens to evade immune clearance. Sustained IL-10 signaling can impair antimicrobial immunity and promote pathogen persistence during chronic infection (12, 55, 56). In experimental models, elevated IL-10 has been associated with impaired pathogen control, including increased persistence of *Mycobacterium tuberculosis* infection, whereas IL-10 deficiency can reduce pathogen burden (3). Clinically, higher IL-10 levels have been linked to chronic viral disease trajectories, including HIV progression and early immune signatures associated with chronic hepatitis C virus infection (57, 58). Some viruses further exploit this pathway by encoding IL-10 homologues; for example, the Epstein–Barr virus BCRF1 gene produces a viral IL-10 that suppresses host immune responses (59).

Mechanistically, IL-10 can inhibit macrophage-mediated clearance of intracellular pathogens and reduce CD8^+^ T-cell responsiveness, thereby contributing to dysfunctional effector states during persistent infection (12). Consistent with this model, IL-10R blockade has been reported to reduce viral persistence in experimental settings (60). These findings illustrate a broader principle that is particularly relevant to cancer: although IL-10-mediated immunoregulation is essential for limiting tissue damage, prolonged or contextually inappropriate signaling can suppress protective immunity and facilitate immune evasion.

## 4 IL-10-mediated enhancement of cytotoxic antitumor immunity: mechanisms and cellular basis

Beyond its well-established anti-inflammatory role, accumulating evidence indicates that IL-10 can promote antitumor immunity in a context-dependent manner (12, 24). Several observations support a protective role for IL-10/IL-10R signaling in selected tumor settings. Individuals carrying loss-of-function mutations in IL-10R have an increased risk of very early-onset diffuse large B-cell lymphoma, while IL-10-/- mice showed heightened susceptibility to inflammation-associated colon cancer (60, 61). Conversely, tumor cells engineered to overexpress IL-10 are often rejected in immunocompetent hosts, whereas their parental counterparts continue to grow (62). Depletion studies further demonstrate that NK cells and CD8^+^ T cells are required for IL-10-dependent tumor control in several mouse models, providing causal evidence that cytotoxic lymphocytes mediate these antitumor effects (62).

These tumor-suppressive effects are not universal. In other settings, particularly those dominated by suppressive myeloid cells, IL-10/IL-10R signaling can promote tumor progression or therapeutic resistance. In multiple myeloma, for example, IL-10R signaling sustains TAM-mediated resistance to proteasome inhibitors, whereas IL-10R blockade reprograms TAMs and restores drug sensitivity (11). IL-10 can also directly blunt CD8^+^ T-cell antigen sensitivity through enhanced N-glycan branching (10). The biological outcome of IL-10 signaling therefore depends on the cellular composition and functional state of the TME.

**Figure 3:**
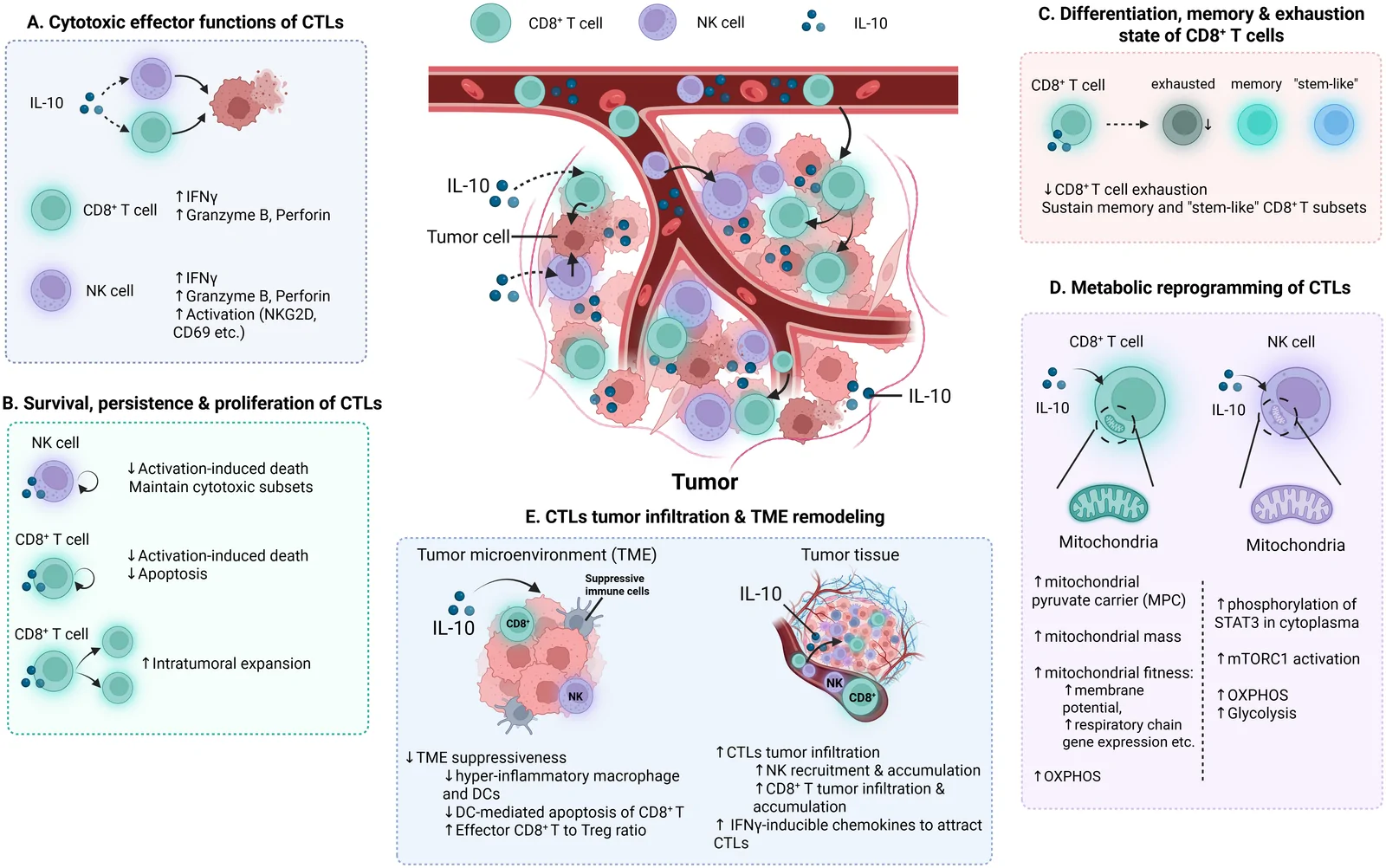
Mechanisms by which IL-10 enhances cytotoxic antitumor immunity. IL-10 can strengthen NK-cell and CD8^+^ T-cell antitumor function through several interconnected mechanisms. **(A)** IL-10 enhances cytotoxic effector function by increasing IFN-γ, granzyme B, and perforin production in NK cells and CD8^+^ T cells and by upregulating NK-cell activation markers. **(B)** IL-10 promotes survival and expansion by limiting activation-induced apoptosis and supporting the intratumoral proliferation of CD8^+^ T cells. **(C)** IL-10 may preserve memory and stem-like CD8^+^ T-cell subsets while restraining progression toward terminal exhaustion, although the evidence supporting these effects is less extensive than that for **(A, B, D)** IL-10 improves the metabolic fitness of cytotoxic lymphocytes by enhancing oxidative phosphorylation (OXPHOS) mitochondrial function, and ATP production. **(E)** IL-10 can remodel the tumor microenvironment (TME) to promote the recruitment, infiltration, and retention of cytotoxic lymphocytes and, in selected contexts, alleviate suppressive myeloid-cell activity. These mechanisms represent the cytotoxic-enhancing effects of IL-10, which are most evident in T-cell-inflamed, lymphocyte-permissive tumors (Section 4.6). The contrasting immunosuppressive effects of IL-10 in myeloid-dominant settings are summarized in **Figure 2**.

This section examines the mechanisms through which IL-10 enhances the antitumor activity of CD8^+^ T cells and NK cells (**Figure 3**). Where relevant, we distinguish causal evidence obtained in genetic and preclinical models from correlative observations in human samples and clinical studies. Tumor contexts in which IL-10R blockade may be preferable are discussed in Section 4.6.

### 4.1 Cytotoxic effector programming

In contrast to its conventional suppressive roles in many innate immune cells, IL-10 can directly enhance the effector program of antigen-experienced CD8^+^ T cells. Following activation, CD8^+^ T cells upregulate IL-10R, increasing their responsiveness to IL-10. In multiple tumor models, delivery of IL-10 through minicircle DNA, recombinant protein, or PEGylated formulations preferentially activate pre-existing tumor-resident CD8^+^ T cells rather than broadly stimulating systemic T-cell populations. IL-10R engagement triggers JAK1/TYK2–STAT3 signaling, promoting local proliferation and marked induction of IFN-γ, granzyme B, and perforin, while CD8^+^ T cells in draining lymph nodes and the spleen are comparatively less affected (4, 6).

Several findings support a direct, intratumoral mechanism. Blocking T-cell trafficking does not abrogate IL-10–induced tumor rejection, indicating that recruitment of newly primed peripheral T cells is not required. Moreover, IL-10Rβ–deficient CD8^+^ T cells fail to expand or acquire the enhanced effector phenotype observed in receptor-competent cells. In these mouse models, direct IL-10R signaling in endogenous CD8^+^ TILs is therefore necessary and, under the experimental conditions tested, sufficient for IL-10-driven tumor control (6).

Human experimental and clinical findings are consistent with this mechanism, although they do not establish causality. *In vitro*, pegylated IL-10 (pegilodecakin) potently augments TCR-driven IFN-γ production and increases granzyme B and perforin expression in human CD8^+^ T cells, with STAT3 acting as a central signaling mediator alongside other pathway inputs (7). In early-phase clinical trials, pegilodecakin treatment was associated with increased systemic IFN-γ and granzyme B, expansion of activated LAG-3^+^PD-1^+^ CD8^+^ T-cell populations, greater intratumoral CD8^+^ T-cell density, and enrichment of cytotoxic and IFN-γ–associated transcriptional signatures in responding patients (9, 63). These associations support the biological plausibility of direct CD8^+^ T-cell activation, but they do not prove that such activation drove clinical responses, particularly because the subsequent randomized phase III trial did not improve survival (23) (Section 5.1 and **Table 1A**). IL-10 has also been reported to enhance proliferation and effector function of activated CD8^+^ T cells from patients with gastric cancer through STAT-dependent signaling (16).

**Table 1A:** Oncology clinical trials involving IL-10-based therapeutics.

Therapeutic format	Name	Clinical trial #	Disease	Stage and reported outcome
PEG-IL-10	Pegilodecakin	NCT02009449	Advanced solid tumors	Phase I, completed — immune activation (CD8^+^ invigoration, IFN-γ/Granzume B induction) reported (8, 9)
		NCT02923921 (SEQUOIA)	Pancreatic cancer	Phase III, completed — did not meet primary endpoint of overall survival; no improvement in progression-free survival (PFS) or objective response rate (ORR) (23)
		NCT03382899	Non-Small Cell Lung Cancer (NSCLC)	Phase II, terminated
		NCT03382912	NSCLC	Phase II, terminated
EGFR-IL-10	DF203	CTR20210299	Solid tumor	Phase I, recruiting
	IAE0972	NCT05396339	Solid tumor	Phase I/II, recruiting
		NCT06719479	Head and Neck Squamous Cell Carcinoma (HNSCC)	Phase II/III, active
B7H3-IL-10	IBB0979	NCT05991583	Solid tumor	Phase I/II, recruiting
		NCT06719479	HNSCC or Nasopharyngeal Carcinoma	Phase II/III, active
Claudin18.2-IL10	LB4330	NCT05707676	Solid tumor	Phase I, terminated
		NCT06468358	Solid tumor	Phase I/II, recruiting
		NCT07177716	Cervical cancer	Phase II/III, active
EGFR-IL-10-IL2	DK210(EGFR)-Deka	NCT05704985	Solid tumor	Phase I, active
IL-10-armored CD19-CAR-NK	CB IL-10/IL15-CD19-CAR NK	NCT06707259	B-cell Non-Hodgkin lymphoma	Phase I, recruiting
IL-10-armored CD19-CAR-T	Meta10-19	NCT05715606	Diffuse Large B-Cell Lymphoma	Investigator-Initiated Trial (IIT)-Phase I, recruiting
		NCT05747157	B-cell Acute Lymphoblastic Leukemia	IIT-Phase I — first-in-human cohort reported high rate of measurable residual disease (MRD)-negative complete remission; predominantly grade 1–2 CRS, no reported ICANS (79)
		NCT06120166	Diffuse Large B-Cell Lymphoma	IIT-Phase I, recruiting
		NCT06277011	B cell lymphoma	IIT-Phase I, recruiting
		NCT06393335	B cell lymphoma	IIT-Phase I, recruiting
		NCT06716164	B-cell Non-Hodgkin lymphoma	IIT-Phase I, recruiting
IL-10-armored TIL	Meta10-19	NCT07106814	Solid tumor	IIT-Phase I, recruiting

**Table 1B:** Non-oncology clinical trials involving IL-10-based therapeutics.

Therapeutic format	Name	Clinical trial #	Disease	Stage and reported outcome
F8-IL-10	Dekavil	NCT02076659	Rheumatoid Arthritis	Phase I, completed
		NCT02270632	Rheumatoid Arthritis	Phase II, terminated
		NCT02711462	Healthy	Phase I, terminated
		NCT03269695	Inflammatory Bowel Disease (IBD)	Phase II, terminated
		NCT03414788	IBD	Phase I, withdrawn
		NCT05622175/NCT07245992	Rheumatoid Arthritis	Phase I, recruiting
Oral gut-selective IL-10	AMT-101	NCT04224857	IBD	Phase I, completed
		NCT04583358	IBD	Phase II, completed
		NCT04741087	Pouchitis	Phase II, completed
		NCT05372939	IBD	Phase II, completed
		EUCTR2020-003955-14	Rheumatoid Arthritis	Phase II, terminated
IL-10-armored Treg	TRX103-Tr1x	NCT06462365	Graft-versus-host disease (GvHD)	Phase I, recruiting
		NCT06721962	Crohn’s disease	Phase I/II, recruiting
IL-10-armored CD19-CAR-T	Meta10-19	NCT06711146	Systemic Lupus Erythematosus	IIT-Phase I, recruiting

IL-10 can likewise act directly on NK cells to enhance activation and cytotoxicity. Across *in vitro* and *in vivo* studies, IL-10 increases NK-cell production of IFN-γ, elevates granzyme B levels, and induces IFN-γ–responsive chemokines such as CXCL10/IP-10, thereby strengthening NK-cell-mediated antitumor responses (15). IL-10 can augment NK-cell killing in a dose-dependent manner, including against tumor targets that are otherwise relatively resistant to NK-cell lysis (5, 64). Moreover, IL-15 stimulation of NK cells can secrete IL-10, which subsequently acts through an autocrine IL-10R–STAT pathway to further enhance cytotoxicity and IFN-γ production (64, 65).

Consistent with these functional effects, IL-10 has been linked to increased expression of NK-cell activation markers, including CD69 and NKG2D, and greater abundance of cytotoxic granule–associated proteins such as TIA-1 (15, 65–68). IL-10 may also indirectly facilitate NK-cell activity by increasing NKG2D ligand expression on target cells, thereby promoting elimination of NKG2D-ligand-high myeloid populations (69). Transcriptional profiling further suggests that IL-10 primarily conditions NK cells for enhanced activation, cytotoxic readiness, and migration rather than inducing broad proliferative expansion (68).

Together, these findings support a model in which IL-10 can directly increase the cytotoxic potential of both CD8^+^ T cells and NK cells. The evidence is strongest in preclinical models, with human experimental and early clinical data providing supportive but largely correlative validation.

### 4.2 Survival, persistence and proliferation

In addition to enhancing effector function, IL-10 can support the survival and persistence of cytotoxic lymphocytes within the TME. In mouse tumor models, IL-10 preserves TIL activity and limits activation-induced cell death, thereby protecting activated CD8^+^ T cells from premature attrition (6). Human correlative studies have similarly linked stronger IL-10/IL-10R signaling with greater CD8^+^ T-cell activity and reduced apoptosis in tumors, including diffuse large B-cell lymphoma, although these associations do not establish a direct causal mechanism (70).

The timing of IL-10 exposure may be particularly important. In preclinical models, delayed or sustained IL-10 delivery after immune priming maintains cytotoxic T-lymphocyte (CTL) function and improves long-term tumor control. These effects have been attributed partly to reduced emergence or activity of suppressive CD4^+^ T-cell populations that would otherwise undermine CTL persistence (6). Such findings suggest that the scheduling of IL-10-based agents relative to priming therapies or checkpoint blockades may be as important as their dose or molecular format.

IL-10 can also promote the proliferation and expansion of anti-tumor CD8^+^ T cells. In several models, IL-10 drives intratumoral CD8^+^ T-cell proliferation (6, 16, 17), whereas genetic disruption of the IL-10R–STAT3 pathway compromises the *in vivo* expansion of activated CD8^+^ T cells (17). Following direct TCR stimulation *in vitro*, IL-10 can partially substitute for IL-2 in supporting CD8^+^ T-cell expansion (54). Consistent with this proliferative capacity, pegylated IL-10 (pegilodecakin) markedly expands tumor-specific CD8^+^ T cells in both transplanted and spontaneous tumors (9, 54). Moreover, depletion of CD4^+^ T-cell does not abrogate pegilodecakin–mediated tumor control in these models (9, 54, 71), supporting the conclusion that tumor rejection is driven predominantly by expanded intratumoral CD8^+^ T cells. This proliferative effect appears to be less prominent in NK cells. In NK cells, IL-10R signaling has been reported to primarily enhance activation and survival without inducing broad proliferative programs, consistent with transcriptional profiling showing limited induction of proliferation-associated genes (69).

Collectively, evidence derived predominantly from genetic and preclinical mouse models indicate that IL-10 can promote CD8^+^ T-cell survival, persistence, and local expansion within tumors. In NK cells, its principal effects appear to involve survival and functional activation rather than proliferation. Whether these mechanisms account for the pharmacodynamic changes observed in human trials remains to be established.

### 4.3 Differentiation, memory, and exhaustion

Beyond its effects on acute cytotoxic function and survival, IL-10 may also influence longer-term CD8^+^ T-cell differentiation, memory formation, and exhaustion. This evidence base is smaller and less independently replicated than that supporting direct effector programming and should therefore be interpreted cautiously.

In infection models, an IL-21/IL-10–STAT3 axis has been implicated in the development of functional memory CD8^+^ T cells. IL-10R–STAT3 signaling supports survival, metabolic fitness, and acquisition of a memory-associated transcriptional program rather than terminal effector differentiation (71, 72). In tumors, IL-10R signaling has similarly been reported to preserve less-differentiated PD-1^int^ TCF-1^+^ stem-like CD8^+^ T-cell population and to associate with durable antitumor immunity and responsiveness to checkpoint blockade in a limited number of preclinical and correlative human studies (17, 19, 73). Conversely, genetic disruption of IL-10R–STAT3 signaling in mouse models skews CD8^+^ T-cell differentiation toward terminally exhausted PD-1^hi^ states with impaired recall capacity (17).

Consistent with these observations, IL-10 can limit apoptosis and delay progression toward terminal dysfunction. *In vitro*, IL-10 enhances the survival of anti-CD3/CD28–activated CD8^+^ T cells, while IL-10 alone or in combination with IFN-γ reduces apoptosis in CD8^+^ T cells from patients with gastric cancer (16). *In vivo*, IL-10R blockade increases the fraction of PD-1^hi^ cells within the CD8^+^ effector compartment. T-cell–specific deletion of IL-10Rβ or STAT3 likewise leads to progressive accumulation of dysfunctional PD-1^hi^ exhausted CD8^+^ T cells, reduced granzyme B production, and impaired immune-synapse formation in genetic mouse models (17).

Human tumor samples provide supporting but correlative evidence. CD8^+^ T cells from IL-10R-deficient patients with chronic lymphocytic leukemia show enrichment of transcriptional signatures associated with terminal differentiation and exhaustion compared with those from IL-10R-proficient patients (17). This finding is consistent with the causal mechanism established in mice but does not independently demonstrate that IL-10R signaling restrains exhaustion in human tumors.

The corresponding role of IL-10 in NK-cell differentiation is less well defined. In infection models, IL-10R signaling protects highly activated NK cells from activation-induced apoptosis and helps maintain preserved cytotoxic subsets (74, 75). However, it remains unclear whether IL-10 maintains a stem-like NK-cell state or directly restrains NK-cell exhaustion in a manner analogous to its proposed effects in CD8^+^ T cells.

### 4.4 Metabolic reprogramming

Chronic antigen stimulation, nutrient depletion, and hypoxia impose substantial metabolic stress on CD8^+^ T cells and NK cells in TME, contributing to exhaustion and loss of cytotoxicity (76). Although IL-10 suppresses inflammatory metabolic programs in many myeloid cells, a smaller but mechanistically detailed body of work indicates that it can instead enhance bioenergetic fitness in cytotoxic lymphocytes. In these cells, IL-10 may function act as a metabolic “adjuvant” that sustains effector activity downstream of IL-10R–STAT3 signaling (5, 19, 77). Because this mechanism has been demonstrated in relatively few model systems, the following findings should be viewed as emerging rather than broadly generalized.

In a defined preclinical model, Guo et al. demonstrated that a half-life–extended IL-10–Fc fusion protein preferentially engage terminally exhausted PD-1^hi^ TIM-3^+^CD8^+^ TILs, which express high levels of IL-10R, and remodels their mitochondrial metabolism (19). IL-10–Fc increases mitochondrial mass, membrane potential, and spare respiratory capacity, and enhanced oxidative phosphorylation (OXPHOS). Mechanistically, it upregulated the mitochondrial pyruvate carrier (MPC) complex and promoted MPC-dependent pyruvate import into mitochondria to fuel OXPHOS. Pharmacological or genetic disruption of mitochondrial pyruvate transport abrogated these effects, whereas exogenous pyruvate partially reproduced them (19, 78).

This metabolic reprogramming restored IFN-γ and TNF-α production, increased granzyme B expression, and promoted local expansion of exhausted CD8^+^ TILs without requiring additional priming in lymphoid organs. These findings provide a mechanistic basis for the synergy observed between IL-10–Fc and PD-1 blockade or adoptive T-cell transfer in the same preclinical models (19).

These metabolic principles have also been applied to adoptive cell therapy. In preclinical solid-tumor models, CAR-T cells engineered to secrete IL-10 preserved mitochondrial integrity, increased MPC-dependent OXPHOS, and showed enhanced proliferation and cytotoxicity within solid tumors, resulting in improved tumor control and features consistent with greater persistence (77). More broadly, because durable T-cell immunity is associated with mitochondrial fitness and oxidative metabolism, the increased respiratory capacity and mitochondrial activity observed in IL-10–stimulated CD8^+^ TILs and IL-10–armored CAR-T cells suggest that IL-10 may promote a more resilient, memory-like bioenergetic state that is less susceptible to exhaustion (19, 77). This interpretation remains a plausible extrapolation rather than an established general property and requires validation across additional tumor models and human tissues. Early clinical studies of IL-10–secreting CAR-T approaches, further suggest that autocrine IL-10 signaling may support *in vivo* expansion and persistence with a manageable toxicity profile (79). These observations are encouraging but remain correlative and do not yet demonstrate the same metabolic mechanism in patients.

IL-10 also reprograms NK-cell metabolism. In human NK cells, IL-10 increases both glycolytic and oxidative metabolism, as reflected by higher extracellular acidification rate and oxygen consumption rate, alongside increased glucose uptake and lactate production (5). Inhibitor studies suggested that IL-10–driven OXPHOS is supported largely by fatty acid oxidation (FAO), with additional input from glycolysis, and that disruption of these pathways reduces IL-10-enhanced effector function (5).

Mechanistically, IL-10 activates STAT3 and downstream mTORC1 signaling, including increased phosphorylation of S6K and S6. Inhibition of either STAT3 or mTORC1 reversed both the metabolic changes and the augmented cytotoxicity, implicating an IL-10R–STAT3–mTORC1 axis in NK-cell metabolic reprogramming (5). Notably, this metabolic enhancement occurs without strong induction of NK-cell proliferation, supporting a primary role in functional optimization rather than numerical expansion.

Collectively, these studies suggest that IL-10 can restore bioenergetic fitness in chronically stimulated CD8^+^ T cells and NK cells, thereby sustaining cytotoxicity in metabolically restrictive tumors. This mechanism may operate in both endogenous TILs and IL-10-armored adoptive cell therapies, but broader replication across tumor models and human tissues is required before it can be considered a general feature of IL-10 biology.

### 4.5 Tumor microenvironment remodeling

IL-10 may enhance cytotoxic immunity not only through cell-intrinsic effects on CD8^+^ T cells and NK cells but also by modifying the TME to relieve extrinsic suppression and promote recruitment, survival, and retention of cytotoxic lymphocytes.

In the J558 plasmacytoma model, IL-10 deficiency skews the TME toward highly suppressive CD4^+^ T-cell and myeloid populations Accumulating inhibitory CD4^+^ T cells constrain CTL-mediated tumor control, whereas restoration of IL-10 signaling or depleting CD4^+^ T cells alleviates this suppression and improves CTL persistence and long-term tumor control (80). In parallel, tumor-targeted IL-10 fusion proteins have also been reported to act on DCs by limiting pathogenic IFN-γ–driven activation and death-ligand expression, thereby reducing DC-mediated apoptosis of CD8^+^ TILs (81).

However, these findings do not imply that IL-10 acts uniformly to relieve myeloid suppression. In myeloid-dominant tumors such as multiple myeloma, IL-10R signaling can instead reinforce TAM-mediated drug resistance, making IL-10R blockade more effective than IL-10 delivery (11). More broadly, modulating IL-10 within the tumor bed can shift the balance between effector and regulatory populations in either direction. In some DC-vaccine and solid-tumor models, reducing IL-10 signaling improves effector-to-Treg ratios and enhances CTL-dependent rejection. In others, controlled, high-dose, or tumor-targeted IL-10 restrains pathogenic inflammatory states in DC or macrophage while preserving, or even improving, the local conditions required for CD8^+^ expansion (9, 26, 82). These apparently divergent findings emphasize that the effect of IL-10 on myeloid cells depends on their activation state and functional role within the tumor.

IL-10 has also been implicated in promoting NK-cell and CD8^+^ T-cell accumulation within tumors. In melanoma metastasis models, systemic IL-10 reduced metastatic burden and was associated with dense NK-cell infiltration of metastatic lesions. NK-cell depletion abolished this protection, indicating that NK cells were causally required in that model (83). Similarly, IL-10 overexpression in gene-modified tumor reduced tumorigenicity and metastasis in an NK-dependent manner, consistent with enhanced NK recruitment/infiltration within the TME (84). IL-10 gene delivery has also been reported to increase CCL5 expression and promote NK-cell trafficking *in vivo* (66, 85).

For CD8^+^ T cells, tumor-cell expression of IL-10 and treatment with recombinant or PEGylated IL-10 have repeatedly increased intratumoral CD8^+^ T-cell numbers, cytotoxic effector signatures, and tumor control in preclinical models. These effects are lost when CD8^+^ T cells are depleted or unable to respond directly to IL-10 (6, 16).

A potential mechanism linking direct lymphocyte activation to TME remodeling is amplification of an IFN-γ–chemokine circuit. IL-10-activated tumor-resident CD8^+^ T cells produce increased IFN-γ, which induces CXCL9/Mig, CXCL10/IP-10, and, in some settings, CCL5 in tumor and stromal cells. These chemokines recruit CXCR3^+^ and CCR5^+^ CD8^+^ T cells and NK cells, creating a feed-forward loop that sustains cytotoxic lymphocyte infiltration and retention (6, 66, 86, 87). Clinical studies of pegylated IL-10 have likewise reported increased intratumoral CD8^+^ T-cell density and enrichment of cytotoxic and IFN-γ-associated gene signatures in responding patients, although these human observations remain correlative (9, 63).

Taken together, IL-10 can remodel lymphocyte-permissive tumors in ways that complement its direct effects on cytotoxic cells. It may attenuate suppressive CD4^+^ T-cell or pathogenic myeloid circuits, protect TILs from deletion, and establish chemokine gradients that support recruitment and retention of CD8^+^ T cells and NK cells. In myeloid-dominant tumors, however, the same pathway may reinforce immunosuppression. IL-10-mediated TME remodeling is therefore bidirectional and highly dependent on cellular context.

### 4.6 Deciding between IL-10 agonism and IL-10R blockade: a context-dependent decision framework

The evidence reviewed in Sections 3–4.5 illustrates that IL-10/IL-10R signaling can support either cytotoxic antitumor immunity or tumor-associated immunosuppression, depending on the tumor and cellular context. **Table 2** synthesizes the tumor and microenvironmental variables discussed above into a working framework for anticipating whether IL-10 agonism or IL-10R blockade is more likely to be beneficial. This framework is derived from mechanistic studies, heterogeneous preclinical models, and correlative patient data; it has not yet been prospectively validated. Testing its predictive value should therefore be a major priority for future studies.

**Table 2:** A context-dependent framework for predicting benefit from IL-10 agonism versus IL-10R blockade.

Variable	Favors IL-10 agonism	Favors IL-10R blockade	Representative evidence
Dominant tumor-immune cell type	T-cell/NK-cell-inflamed tumor	Myeloid (TAM)-dominant tumor	(6, 9, 11)
IL-10R expression on CD8^+^ TILs vs. TAMs/DCs	Higher on CD8^+^ TILs	Higher on TAMs/DCs	(6, 11, 19)
Baseline systemic/intratumoral IL-10	Low-to-moderate	Already elevated	(11, 12)
Treg/TAM abundance	Low	High	(11, 80)
Tumor antigenicity/mutational burden	Higher (antigen-experienced TILs present)	Lower/poorly antigenic	(6, 9)
IFN-γ pathway responsiveness	Intact	Impaired	(6, 66, 86, 87)
TIL exhaustion/metabolic state	Stem-like PD-1int TCF-1^+^ pool present	Predominantly terminally exhausted or metabolically collapsed	(17, 19, 73)
Disease context (illustrative)	T-cell-inflamed solid tumors (e.g., NSCLC, renal cell carcinoma (RCC), melanoma cohorts in early trials)	Myeloid-driven hematologic malignancy (e.g., multiple myeloma)	(8, 9, 11)

Broadly, the available evidence favors IL-10 agonism in tumors that are already antigenic and T-cell inflamed, contain appreciable numbers of IL-10R-expressing CD8^+^ TILs or NK cells, retain functional IFN-γ signaling, and have a comparatively limited burden of suppressive TAMs and Tregs. These features resemble the settings, in which pegilodecakin and IL-10–Fc produced the clearest evidence of CD8^+^ T-cell activation and metabolic reinvigoration (4, 6, 9, 19, 63).

By contrast, IL-10R blockade, or therapeutic strategies that avoid broad IL-10 exposure, may be preferable in myeloid-dominant, TAM-rich tumors with high baseline IL-10 activity and limited cytotoxic lymphocyte infiltration. Multiple myeloma provides a representative example, in which IL-10R blockade reprogrammed TAMs and restored sensitivity to proteasome inhibition (11).

Many tumors are unlikely to fall neatly into either category. In these intermediate contexts, tumor-targeted or cell-selective IL-10 formats may offer a rational alternative to systemic agonism or systemic blockade. Such approaches aim to concentrate IL-10 activity on cytotoxic lymphocytes while limiting exposure of suppressive myeloid or regulatory populations. The optimal strategy should therefore be determined not simply by the presence of IL-10, but by identifying which cell populations receive and dominate the biological response to IL-10R signaling within each tumor.

## 5 IL-10–based cancer immunotherapy: engineering strategies and translational approaches

As understanding of IL-10 biology has evolved, so too has interest in its therapeutic application in cancer. Although traditionally viewed as an anti-inflammatory cytokine, can enhance cytotoxic immunity through direct effects on CD8^+^ T cells and NK cells. This activity has created opportunities to treat immune-resistant tumors, but it also requires careful contextual targeting because IL-10 can reinforce immunosuppressive myeloid programs in other settings, as discussed in Section 4.6.

**Figure 4:**
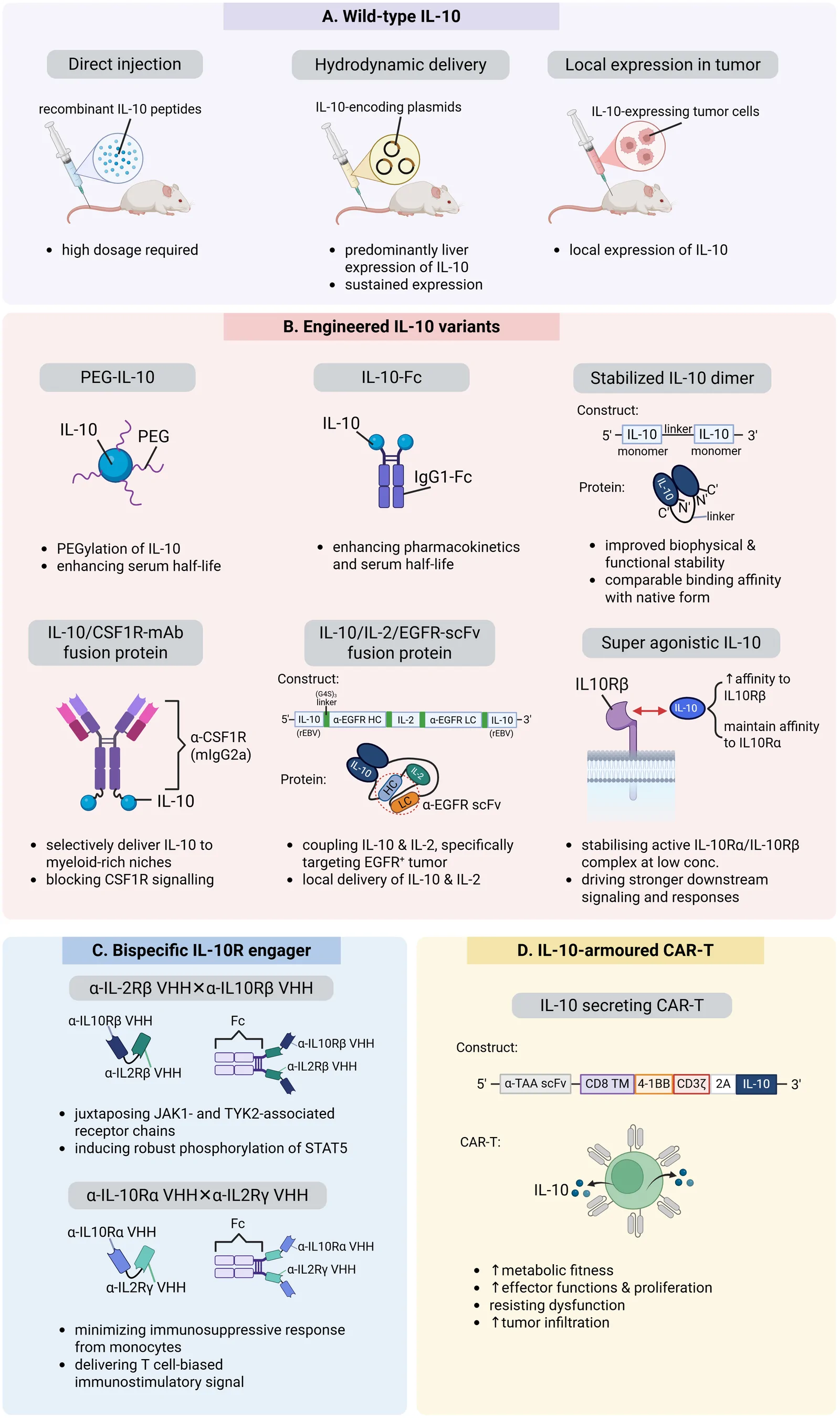
Current cancer immunotherapy strategies harnessing IL-10. Multiple approaches have been developed to exploit the antitumor potential of IL-10. **(A)** Early studies evaluated wild-type IL-10 through direct administration of recombinant protein, hydrodynamic delivery of IL-10-encoding plasmids, or engineered IL-10-secreting tumor cells. **(B)** Subsequent protein engineering strategies have extended IL-10 half-life through PEGylation or Fc fusion, improved its stability using covalently stabilized dimers, concentrated its activity through antibody- or scFv-based targeting modules, and enhanced receptor engagement by increasing receptor-binding affinity. **(C)** Bispecific surrogate agonists use paired nanobodies to juxtapose selected IL-10R- and IL-2R-associated receptor chains, thereby redirecting JAK–STAT signaling and enabling control over receptor geometry, valency, and signaling output. **(D)** CAR-T cells engineered to secrete IL-10 display improved metabolic fitness, effector function, proliferation, tumor infiltration, and resistance to dysfunction. Clinical translation of these platforms remains at an early stage: most approaches shown are preclinical, and the only randomized phase III trial of an IL-10-based oncology agent, pegilodecakin, did not meet its primary efficacy endpoint (Section 5; **Table 3**).

A range of therapeutic strategies has therefore been developed to improve the pharmacokinetics, localization, receptor engagement, and cellular selectivity of IL-10 activity. These include pharmacokinetically optimized wild-type IL-10, engineered IL-10 variants, tumor- and cell-targeted fusion proteins, bispecific IL-10R engagers, and IL-10–armored adoptive cell therapies (**Figure 4**; **Table 3**). Several of these approaches have entered early-phase clinical testing, either as monotherapies or in combination regimens, and have shown evidence of on-target immune activation and preliminary antitumor activity in selected settings (**Table 1A**). However, the only randomized phase III oncology trial completed to date did not meet its primary efficacy endpoint (46). Thus, although IL-10 represents an increasingly engineerable component of cancer immunotherapy, its clinical development requires adequately powered efficacy studies and rigorous evaluation of the safety considerations discussed in Section 5.5.

**Table 3:** Comparison of IL-10 engineering and delivery platforms discussed in Section 5.

Platform		Mechanism	Primary target cell(s)	Tumor localization strategy	Signaling bias	Development stage	Key efficacy data	Main safety risks	Open questions
PEGylated IL-10 (pegilodecakin)		Half-life extension via PEGylation	CD8^+^ TILs (preferential)	None (systemic; relies on preferential TIL activation)	STAT3-dominant	Phase III completed (negative); multiple Phase I/II	CD8^+^ invigoration, IFN-γ/Granzyme B induction in early trials (7–9); SEQUOIA phase III negative for OS/PFS/ORR (23)	Dose-dependent thrombocytopenia, anemia, neutropenia, fatigue (8, 23)	Why pharmacodynamic activation did not translate to survival benefit
IL-10–Fc fusion		FcRn-mediated half-life extension	Terminally exhausted PD-1^hi^ TIM-3^+^ CD8^+^ TILs	Preferential (high IL-10R on target TILs)	STAT3-dominant	Preclinical	Metabolic reprogramming (MPC/OXPHOS), synergy with PD-1 blockade and ACT in mouse models (19)	Not yet tested clinically	Generalizability across tumor models; human translation
Covalently stabilized IL-10 dimer		Engineered linker locks dimer conformation	Broad (IL-10R^+^ cells)	None (stability/manufacturing improvement)	STAT3-dominant	Preclinical (validated mainly in inflammatory models)	Improved biophysical stability; retained STAT3 signaling (90)	Not yet characterized in oncology setting	Oncology efficacy data; need for tumor-targeting add-on
Myeloid-targeted fusion (e.g., α-CSF1R–IL-10, BF10)	IL-10 delivery + CSF1R blockade		TAMs (delivery), CD8^+^ T cells (functional effect)	Targeted via CSF1R^+^ myeloid cells	STAT3-dominant	Preclinical	Reduced TAM/Treg, increased CD8+ infiltration, PD-1 combo benefit in mouse models (21)	Not yet tested clinically; on-target myeloid depletion effects unknown in humans	Clinical safety/efficacy; applicability beyond CSF1R-high tumors
Tumor-targeted immunocytokine (e.g., EGFR-IL-2/IL-10)	Tumor-targeted co-delivery of IL-2 and IL-10		CD8^+^ T cells within EGFR^+^ tumors	EGFR-targeted scFv	STAT3 + IL-2R signaling	Preclinical	Reduced cytokine-storm signature vs. IL-2 alone; CD8^+^ activation/infiltration in mouse models (20)	Not yet tested clinically; on-target/off-tumor EGFR effects unknown	Human PK/safety; applicability to EGFR-low tumors
IL-10Rβ super-agonist	Affinity-enhanced IL-10Rβ engagement		Myeloid cells and CD8^+^ T cells	None (potency/dose-sparing strategy)	STAT1/STAT3	Preclinical	Lower-dose activity than WT IL-10; enhances CAR-T cytolytic activity with IL-2 (91)	Not yet characterized; lower dosing may reduce systemic exposure but unproven	Whether dose-sparing improves therapeutic index *in vivo*
Bispecific VHH IL-2Rβ×IL-10Rβ engager	Forced receptor-chain co-engagement		T cells, NK cells	None (systemic; cell-type selectivity via receptor pairing)	STAT5-dominant (non-native)	Preclinical	STAT5 activation, proliferation, cytotoxicity in human T/NK cells (45)	Unknown; STAT5-biased signaling safety profile uncharacterized	*In vivo* tumor efficacy; safety of non-native signaling bias
Bispecific VHH IL-10Rα×IL-2Rγ engager	Forced receptor-chain co-engagement		CD8^+^ T cells	None (systemic)	STAT3-dominant (hybrid)	Preclinical	IFN-γ/GzB induction and CD8^+^ expansion comparable to PEG-IL-10 (22)	Unknown	*In vivo* tumor efficacy; comparative safety vs. PEG-IL-10
IL-10-armored CAR-T (e.g., META10-19)	Cell-intrinsic constitutive IL-10 secretion		Engineered CAR-T cells (autocrine/paracrine)	Local (tumor-bed secretion by infused cells)	STAT3-dominant	Phase I (hematologic; first-in-human)	High MRD-negative CR rate, robust *in vivo* expansion in B-ALL (77, 79)	Predominantly grade 1–2 CRS, no reported ICANS in the single reported cohort (79); bystander/off-tumor effects unaddressed	Performance in solid tumors; larger/controlled safety datasets; antigen-loss relapse risk

### 5.1 Wild-type IL-10

Early translational efforts established proof-of-concept that wild-type IL-10 can suppress tumor growth and metastasis *in vivo*, in part through recruiting and activating NK cells at tumor sites (87). Clinical administration of recombinant IL-10 is nevertheless constrained by its short circulating half-life, which is less than 5 hours in humans, and by the high doses required to sustain biologically meaningful exposure (45).

Hydrodynamic plasmid delivery, which achieves sustained hepatic IL-10 expression at approximately 10–100 ng/ml for up to 20 days, demonstrated that prolonged systemic IL-10 exposure could induce tumor regression and NK-cell accumulation in mouse models (6, 69). Similarly, local IL-10 expression by genetically modified tumor cells reduced tumor growth and invasiveness in immunocompetent hosts (92). Although neither delivery strategy progressed into clinical development, these studies established the biological plausibility of IL-10 therapy and provided a pharmacokinetic rationale for the half-life extension and targeted-delivery approaches described below. Subsequent experience with pegylated IL-10, however, also illustrates the gap between preclinical activity and demonstrated clinical benefit.

### 5.2 Engineered IL-10 variants

To improve the short exposure window and off-target immunoregulatory effects of wild-type IL-10, multiple engineered formats have been developed to extend systemic persistence, tune receptor engagement, improve molecular stability, or concentrate activity within tumors. These include PEGylated IL-10, IL-10-Fc fusions, covalently stabilized dimers, receptor-affinity–enhanced “super-agonists”, and multifunctional fusion proteins that couple IL-10 incorporating tumor- or cell-targeting modules. Collectively, these approaches aim to increase the magnitude and durability of IL-10–driven cytotoxic immunity while improving therapeutic selectivity. **Table 3** compares these platforms, together with the surrogate receptor agonists and armored cell therapies described in Sections 5.3 and 5.4, according to their mechanism, target cells, localization strategy, developmental stage, and principal safety considerations.

PEGylated IL-10 (pegilodecakin). PEGylation increases the hydrodynamic radius of IL-10, reduces renal clearance, and improves proteolytic stability, thereby prolonging systemic exposure. In preclinical tumor models, PEG–IL-10 selectively enhanced cytotoxic programs in intratumoral CD8^+^ T cells and promoted tumor control (4). Translational studies subsequently showed that pegilodecakin amplifies TCR-driven IFN-γ production and induces perforin and granzyme B expression in CD8^+^ T cells (7).

In early-phase clinical trials, pegilodecakin induced pharmacodynamic changes consistent with systemic immune activation, including expansion of activated CD8^+^ T-cell populations and intratumoral immune changes associated with responses in selected settings (8, 9). Combination regimens with PD-1 blockade the phase 1b IVY study further supported the feasibility of using pegilodecakin as a CD8^+^ T-cell–invigorating partner, with manageable toxicity in early-phase setting (88). In murine models, PEG–IL-10 also shifted intratumoral CD4^+^ T-cell composition away from Treg dominance and toward a more effector-permissive environment, providing an additional rationale for combination with checkpoint blockade (89).

Despite these pharmacodynamic findings, the randomized phase III SEQUOIA trial of pegilodecakin plus FOLFOX in gemcitabine-refractory pancreatic cancer did not improve overall survival, progression-free survival, or objective response rate compared with FOLFOX alone (46). Exploratory analyses nevertheless showed increases in IFN-γ, granzyme B, and IL-18, consistent with on-target immune activation. This dissociation between pharmacodynamic activity and clinical efficacy is a major cautionary finding for the field and is discussed further in Section 6.

IL-10–Fc fusion proteins. Fusion to an Fc domain extends IL-10 half-life through FcRn-mediated recycling and enables sustained IL-10R engagement *in vivo*. In solid-tumor models, a cross-reactive IL-10–Fc fusion preferentially acted on terminally exhausted PD-1^hi^TIM-3^+^ CD8^+^ TILs, expanding this subset and restoring IFN-γ, TNF, granzyme B production (19). As discussed above in Section 4, these effects were linked to IL-10–Fc–driven metabolic reprogramming toward MPC-dependent OXPHOS.

IL-10–Fc also enhanced responses to adoptive T-cell transfer and PD-1 blockade in preclinical models (19). This format may therefore provide a means of revitalizing metabolically constrained, terminally exhausted TILs. However, confirmation in independent tumor models and subsequent clinical evaluation will be required to establish the generalizability and therapeutic relevance of these findings.

Covalently stabilized IL-10 dimers. Native IL-10 forms a non-covalently associated dimer that can be susceptible to dissociation and instability. Head-to-tail covalently linked IL-10 dimers have therefore been engineered using flexible peptide linkers to stabilize the active dimeric conformation (90). These constructs retain canonical IL-10R signaling, including STAT3 activation, while showing improved biophysical stability under pH, temperature stress and storage. Although their initial *in vivo* evaluation was largely performed in inflammatory models, such stabilized dimers may provide useful scaffolds for oncology, particularly when combined with tumor- or cell-targeted delivery.

Myeloid-targeted IL-10 fusion proteins. Systemic IL-10 presents a particular challenge in tumors in which IL-10R signaling sustains suppressive TAM programs. In multiple myeloma, for example, IL-10R blockade reprogrammed TAMs, restored sensitivity to proteasome inhibitors, and reversed drug resistance (11). This finding illustrates why IL-10 delivery must be designed around the dominant responding cell type rather than assumed to be uniformly beneficial.

One approach is to couple IL-10 delivery to simultaneous disruption of a suppressive myeloid pathway. BF10 is an anti-CSF1R–IL-10 fusion protein engineered to target IL-10 to CSF1R^+^ compartments while blocking CSF1R signaling (21). The molecule comprises an anti-CSF1R IgG linked to an IL-10 payload and retains both CSF1R binding and IL-10R–STAT3 signaling. In preclinical tumor models, BF10 outperformed either IL-10 or anti-CSF1R monotherapy, reducing suppressive TAM and Treg populations, increasing CD8^+^ T-cell infiltration and expansion, and improving responsiveness to PD-1 blockade (21). These findings illustrate how a targeted fusion architecture can reconcile IL-10 delivery with simultaneous control of myeloid-mediated suppression.

Tumor-targeted IL-10–based immunocytokines. Tumor-targeted immunocytokines seek to concentrate IL-10 activity within the TME while limiting systemic exposure. One example is an EGFR-targeted IL-2/IL-10 immunocytokine containing a split anti-EGFR scFv, with IL-2 inserted between its VH and VL domains and two monomeric EBV IL-10 M2 muteins positioned to form a dimeric IL-10 payload (20). This architecture preferentially binds EGFR^+^ tumor cells and locally presents IL-2 and IL-10 to infiltrating immune cells.

Within this construct, IL-10 is proposed to moderate IL-2-associated inflammatory toxicity, whereas IL-2 enhances IL-10-mediated CD8^+^ T-cell activation, proliferation, and cytotoxicity. In murine tumor models, the fusion reduced cytokine storm-like inflammatory signatures while increasing intratumoral CD8^+^ T-cell activation, infiltration, and tumor-cell killing. Combination with PD-1 blockade further improved tumor control (20). This approach illustrates how tumor targeting and complementary cytokine functions can be integrated within a single molecule to improve therapeutic index.

Super agonistic IL-10 variant. A complementary engineering strategy is to increase IL-10 affinity for IL-10Rβ, the lower-affinity signaling subunit, thereby stabilizing assembly of the active IL-10Rα/IL-10Rβ complex at low ligand concentrations (91). Using yeast surface display and structure-guided mutagenesis, Gorby et al. optimized residues at the IL-10- IL-10Rβ interface while preserving IL-10Rα binding.

Compared with wild-type IL-10, the resulting IL-10Rβ–affinity–enhanced variant induced IL-10–responsive gene expression at substantially lower concentrations and produced stronger STAT1 and STAT3 phosphorylation in human myeloid cells and CD8^+^ T cells. It also increased granzyme B expression in primary human CD8^+^ T cells and, in combination with IL-2, enhanced the cytolytic activity of tumor-specific CAR-T cells relative to wild-type IL-10 supplementation (91). These findings suggest that increasing IL-10Rβ affinity can generate a low-dose super-agonist with enhanced capacity to program cytotoxic T cells. Its therapeutic value will nevertheless depend on whether increased potency can be achieved without proportionally increasing off-target or systemic effects.

### 5.3 Bispecific IL-10R engagers as surrogate cytokine agonists

Beyond modifying the native cytokine, IL-10R-associated signaling can be reconstructed using bispecific surrogate agonists. These synthetic molecules physically juxtapose selected receptor chains, allowing signaling geometry, receptor pairing, valency, and potentially cell-type selectivity to be controlled more precisely than with native IL-10 (45).

One example is a VHH-based IL-2Rβ×IL-10Rβ surrogate agonist generated by linking an anti-IL-2Rβ VHH to an anti-IL-10Rβ VHH within a single polypeptide (45). This compact bispecific molecule, approximately 30–35 kDa in size, co-engages IL-2Rβ and IL-10Rβ, which are expressed on T cells and NK cells. Bringing these receptor chains together juxtaposes their associated kinases and initiates JAK–STAT signaling. As discussed in Section 2, this engineered receptor pairing predominantly activates STAT5 rather than the STAT3-dominant output of the native IL-10 receptor complex. In human T cells and NK cells, the construct induced robust STAT5 phosphorylation, proliferation, and cytotoxic activity (45). Fc-fused versions further increased functional potency and systemic exposure, likely through enhanced avidity, increased valency, and prolonged half-life (45).

A complementary design is a VHH-based IL-10Rα×IL-2Rγ surrogate agonist, created by linking an anti–IL-10Rα VHH to an anti–common γ-chain (IL-2Rγ) VHH (22). By juxtaposing these receptor subunits, the molecule generates a hybrid signaling response in CD8^+^ T cells that includes STAT3 phosphorylation, increased IFN-γ and granzyme B production, and CD8^+^ T-cell expansion. In the reported experimental setting, these effects were comparable to those induced by PEG–IL-10 (22). Fc-fused variants provide an additional means of tuning exposure, valency, and functional potency.

Together, these molecules demonstrate that IL-10R-associated signaling can be reconstructed and redirected through defined receptor-chain combinations. Their development also reinforces the principle that biological output depends not simply on engagement of IL-10Rα, but on the receptor-associated kinases and STAT pathways brought into proximity. Important next steps include evaluating these molecules in tumor-bearing models, determining their cellular selectivity and signaling durability, and assessing their compatibility with checkpoint blockade and adoptive cell therapies. Because some constructs are biased toward STAT5 rather than native STAT3-dominant signaling, their efficacy and toxicity profiles may also differ fundamentally from those of conventional IL-10 formats.

### 5.4 IL-10-armored adoptive immune cell therapy

Rather than delivering IL-10 systemically, adoptive immune cells can be engineered to produce it locally at sites of antigen recognition. This strategy has been applied most extensively to CAR-T cells. In a prototypical design, a secreted IL-10 transgene is incorporated into the same viral vector as the CAR, in a multicistronic cassette separated by a self-cleaving 2A peptide (77). A shared promoter coordinates CAR and IL-10 expression, while a signal peptide directs IL-10 secretion. Each CAR^+^ T cell thereby becomes a local source of IL-10 within the tumor, potentially maximizing autocrine and paracrine activity while limiting the need for high systemic cytokine exposure.

In this setting, IL-10 is intended to function primarily as a local metabolic and functional support signal. A key preclinical study showed that constitutive IL-10 secretion enabled CAR-T cells to resist dysfunction and mediate durable clearance of primary and metastatic lesions across multiple solid-tumor models (77). IL-10–armored CAR-T cells displayed reduced exhaustion, enhanced proliferation and cytotoxicity under chronic antigen exposure, and preserved polyfunctional cytokine production and granzyme B expression. They also showed increased tumor infiltration and features associated with stem-like memory, supporting sustained persistence and protection against tumor rechallenge.

This concept has begun to enter clinical evaluation. META10-19, an IL-10–secreting anti-CD19 CAR-T product, produced high rates of minimal residual disease-negative complete remission in a first-in-human phase I study of relapsed or refractory B-cell acute lymphoblastic leukemia following a single low-dose infusion (79). Notably, most cytokine release syndrome (CRS) events were grade 1 or 2, and no immune effector cell-associated neurotoxicity syndrome (ICANS) was reported. The product also underwent substantial *in vivo* expansion that correlated with serum IL-10 levels, a pharmacokinetic/pharmacodynamic association consistent with autocrine IL-10 support of CAR-T-cell fitness and persistence.

These findings provide early clinical support for the feasibility of IL-10 armoring but should be interpreted cautiously. The study was small, single-arm, and conducted in one hematologic malignancy; it therefore cannot establish that IL-10 secretion caused the observed responses or exclude contributions from other product- or patient-related factors. Larger and controlled studies will be required before this strategy can be extended confidently to other hematologic malignancies or solid tumors.

### 5.5 Safety and toxicity considerations

As with any cytokine-based immunotherapy, the translational benefits of IL-10-based strategies must be weighed against safety liabilities that vary according to whether IL-10 is administered systemically, incorporated into an engineered protein, or secreted locally by adoptive immune cells.

Systemic IL-10 and engineered variants. Recombinant and pegylated IL-10 produce measurable, dose-dependent hematologic toxicity in humans. In a phase I dose-escalation study of pegilodecakin monotherapy in advanced solid tumors (AM0010), dose-limiting toxicities included thrombocytopenia and anemia, consistent with the known effects of IL-10 on hematopoietic progenitors (8). In the phase III SEQUOIA trial, grade ≥3 adverse events occurring at least 5% more frequently with pegilodecakin plus FOLFOX than with FOLFOX alone included thrombocytopenia (25.2% vs. 3.6%), anemia (16.2% vs. 4.0%), neutropenia (29.5% vs. 22.7%), and fatigue (17.6% vs. 10.8%) (23). These findings demonstrate that systemic IL-10 exposure at doses sufficient to induce immune activation can impose a substantial hematologic burden. Future engineered formats must therefore show not only pharmacodynamic activity but also a meaningful improvement in therapeutic index.

IL-10’s canonical suppression of antigen-presenting-cell function raises an additional concern that sustained or poorly targeted systemic exposure could impair antimicrobial immunity and increase susceptibility to infection. Published IL-10 oncology studies have not, to our knowledge, comprehensively quantified or mechanistically characterized this risk, making infection-related outcomes an important priority for future safety reporting. Similarly, in myeloid-dominant tumors, broad IL-10 exposure could reinforce TAM- or Treg-mediated suppression rather than promote cytotoxic immunity. This possibility provides a major rationale for the tumor-targeted, cell-targeted, and multifunctional fusion strategies described in Section 5.2.

IL-10-armored adoptive cell therapies. For CAR-T, CAR-NK, and TIL products engineered to secrete IL-10 locally, the primary safety questions differ from those associated with systemic protein administration. These include whether local IL-10 secretion modifies the incidence or severity of CRS/ICANS, produces bystander suppression of non-target immune cells, alters the magnitude or duration of cellular persistence, or influences the risk of antigen-loss relapses.

The limited clinical evidence available is encouraging but insufficient to resolve these questions. In the first-in-human META10–19 study, most cytokine release syndrome events were grade 1 or 2, and no ICANS was reported after a single low-dose infusion (79). These findings suggest that IL-10 secretion at the doses tested did not produce severe CRS or ICANS. However, they derive from a single early-phase, single-arm study in relapsed or refractory B-cell acute lymphoblastic leukemia.

Several safety issues therefore remain unresolved. It is not yet known whether IL-10-armored products suppress adjacent non-target immune populations *in vivo*, whether enhanced persistence increases selective pressure for antigen-loss escape, or how these therapies behave in solid tumors with more complex myeloid and stromal compartments. These questions should be evaluated prospectively in larger and more diverse cohorts rather than assumed to be resolved by the favorable early toxicity profile. Safety assessment must therefore develop alongside efficacy testing as an integral component of IL-10-based therapeutic engineering.

## 6 Conclusions and future directions

The evidence reviewed here supports a coherent, albeit initially counterintuitive, view of IL-10 biology in cancer. Although IL-10 is classically defined by its capacity to suppress inflammation, under specific conditions, it can enhance cytotoxic antitumor immunity. In other settings, particularly in myeloid-dominant tumors, the same cytokine can reinforce the immunosuppressive programs for which it is traditionally known. Across experimental and early clinical studies, IL-10 has been reported to promote CD8^+^ T-cell and NK-cell effector programming, support cytotoxic lymphocyte survival and persistence, restrain terminal exhaustion, reprogram cellular metabolism to withstand the nutrient-depleted and hypoxic TME, and facilitate the infiltration and retention of cytotoxic effectors. A substantial body of preclinical and correlative human evidence attributes these effects to direct IL-10R–STAT3 signaling in antigen-experienced cytotoxic lymphocytes, with mechanistic insights now extending from transcriptional regulation to mitochondrial bioenergetics. Nevertheless, as emphasized throughout Section 4, several of these proposed mechanisms remain supported by only a limited number of model systems, and their clinical relevance has yet to be firmly established.

Reconciling these cytotoxicity-enhancing effects with IL-10’s canonical immunosuppressive functions requires consideration of the cellular and signaling context. Section 2 therefore proposes a combinatorial signaling model — encompassing STAT1, STAT3, and STAT5 engagement; mTORC1 signaling; NF-κB and AP-1 cooperation; receptor density; feedback regulation; and chromatin state — that, in our view, explains this functional duality more effectively than a single shared signaling axis. Although IL-10 signals through a common receptor complex and predominantly activates the JAK1/TYK2–STAT3 pathway across cell types, the downstream biological outcomes are shaped by the identity and activation state of the responding cell, the additional pathways engaged, and the surrounding microenvironment.

In chronic inflammatory settings and myeloid-dominant tumors, IL-10 acts primarily on macrophages. dendritic cells, TAMs, suppressing pro-inflammatory cytokine production, limiting tissue damage, or, in some tumors, reinforcing drug-resistant TAM programs (11). In lymphocyte-permissive tumors, however, the same core signaling machinery can instead operate on activated, antigen-experienced CD8^+^ TILs and NK cells. In these cells, STAT3 activation, in cooperation with NF-κB and AP-1, could promote metabolic fitness, cytotoxic effector expression, and resistance to terminal exhaustion. IL-10 is therefore best viewed as neither exclusively immunosuppressive nor immunostimulatory, but as a context-dependent regulator of immune homeostasis. Its therapeutic manipulation must accordingly be matched to the tumor–immune context, whether through agonism, targeted delivery, or blockade, as outlined in the decision framework in Section 4.6.

Translating this context-dependent biology into clinically effective therapeutics represents both the central challenge and a major opportunity for the field. Multiple engineering strategies have demonstrated that IL-10 activity can be spatially and temporally controlled through half-life extension, enforced receptor affinity, tumor-targeted fusion architectures, inducible expression systems, and cellular armoring of adoptive therapies (**Table 2**). Early clinical studies support the feasibility of several of these approaches and provide an initial pharmacodynamic framework. Reported changes include the expansion of activated granzyme B^+^ CD8^+^ T-cell populations, induction of intratumoral IFN-γ/CXCL9/CXCL10 signatures, and, for IL-10–armored CAR-T cells, autocrine-driven *in vivo* expansion associated with serum IL-10 levels.

These pharmacodynamic findings, however, should not yet be interpreted as reliable predictors of clinical benefit. In the randomized phase III SEQUOIA trial, the expected immune-activation biomarker changes were observed without a corresponding improvement in survival (46). This dissociation highlights the limitations of using pharmacodynamic biomarkers as surrogate endpoints before they have been formally validated against clinical outcomes. Identifying the patient populations and tumor contexts most likely to benefit — and prospectively testing, rather than assuming, the relevant biomarker hypotheses— therefore remain a central priority.

Four interrelated design and validation principles emerge from the mechanistic and translational evidence reviewed here. First, spatiotemporal control of IL-10 delivery will be critical. Tumor-targeted formats, carefully designed myeloid-targeted approaches that avoid reinforcing TAM suppression, inducible expression systems, and locally secreting adoptive cells all offer strategies for maximizing intratumoral IL-10 activity while limiting systemic immunomodulation and its associated hematologic and infectious risks (Section 5.5). Preclinical findings on dose and timing provide an initial framework for therapeutic scheduling. Particularly, delayed or sustained IL-10 delivery after immune priming may be advantageous, as it may preferentially engage terminally exhausted PD-1^hi^TIM-3^^+^^ TILs with high IL-10R expression. These observations support combining IL-10–based agents with priming therapies or checkpoint blockade, but the proposed scheduling principles require prospective validation.

Second, rational combination with complementary immunotherapies will likely be needed to maximize IL-10’s antitumor effects while limiting its risks. Combining IL-10–based agents with PD-1/PD-L1 blockade is supported by mechanistic evidence that can improve the metabolic fitness and effector capacity of exhausted TILs. In tumors with prominent myeloid-mediated suppression, combinations with myeloid-modulating agents, such as anti-CSF1R or anti-CD47 therapies, may help prevent IL-10 signaling from reinforcing immunosuppressive TAM programs. The choice of combinations should therefore be guided by the dominant immune constraints within each tumor context, as formalized in the decision framework in Section 4.6.

Third, biomarker-guided patient selection will be essential, although the candidate biomarkers discussed here should be regarded as hypotheses for prospective evaluation rather than validated selection tools. Potential biomarkers include the relative abundance and expression of IL-10R on tumor-infiltrating CD8^+^ T cells versus TAMs and DCs; the balance between stem-like PD-1^int^TCF-1^+^ and terminally exhausted PD-1^hi^ TIM-3^+^ subsets within the TIL subsets; circulating and intratumoral levels of IL-10, IFN-γ, and CXCL9, and CXCL10; exhaustion-associated transcriptional markers such as TCF-1 and TOX, interpreted alongside surface expression of PD-1, TIM-3, and LAG-3; and metabolic indicators of mitochondrial fitness, including mitochondrial mass, membrane potential, OXPHOS-associated gene signatures, and MPC expression. Because no single marker is likely to capture the complexity of IL-10 responsiveness, composite biomarkers integrating cellular composition, receptor distribution, functional state, and metabolic fitness may ultimately prove more informative.

Fourth, safety must be incorporated into therapeutic design and assessed with the same rigor as efficacy from the earliest stages of clinical development. For recombinant proteins and engineered IL-10 variants, evaluation should include systemic hematologic and infectious toxicities, as well as the consequences of prolonged or off-tumor immune modulation. For IL-10–armored cell therapies, prospective evaluation should also address bystander immunosuppression, the risks of CRS and ICANS, uncontrolled or excessive cellular expansion, and the potential for antigen-loss relapse. in larger and more diverse cohorts, and the potential for antigen-loss relapse. These risks must be evaluated in larger and more diverse cohorts rather than inferred from small scale early-phase studies. Safety should therefore be treated as an integral component of platform design, dose selection, and patient stratification, rather than as a secondary consideration once efficacy signals emerge.

Taken together, the mechanistic advances and translational progress reviewed here position IL-10 not as a peripheral cytokine of uncertain therapeutic value, but as a biologically grounded and increasingly engineerable axis for restoring cytotoxic fitness in the immunosuppressive TME. Its therapeutic potential, however, depends on explicitly accounting for—and designing around—its capacity to produce both beneficial and harmful immune effects. IL-10–based platforms remain at an early stage of clinical validation across tumor types, with one negative randomized phase III trial and a broader body of early-phase, uncontrolled, and preclinical evidence supporting continued investigation. By advancing targeted delivery, rational combination, biomarker-guided deployment, and rigorous safety assessment, these platforms may ultimately convert metabolically fragile, short-lived anti-tumor immunity into durable clinical responses. Realizing that potential will require prospective, adequately powered trials that test directly, rather than assume, the mechanistic hypotheses reviewed here.
